# Casting the Net Widely for Change in Animal Welfare: The Plight of Birds in Zoos, Ex Situ Conservation, and Conservation Fieldwork

**DOI:** 10.3390/ani12010031

**Published:** 2021-12-23

**Authors:** Gisela Kaplan

**Affiliations:** School of Science and Technology, University of New England, Armidale, NSW 2351, Australia; gkaplan@une.edu.au

**Keywords:** avian welfare, bird behavior, pain, fear, learning, invasive procedures, telemetry, data collection, conservation, compassion, validity of research results

## Abstract

**Simple Summary:**

Animal welfare measures have been designed to improve the health and environmental conditions of animals living under human control, for whatever reason. Welfare regulations have evolved also in line with new research insights into the cognitive, affective, and physiological domain of birds, as this paper discusses. This paper casts a critical eye on areas that Animal Welfare regulations have not reached at all, have not gone far enough, or are not regulated or supervised. It identifies the plight of birds living in captivity or being studied in the field, which either by neglect, ignorance, or design are subject to practices and procedures that may not meet basic welfare standards. The paper discusses some profound contradictions in the way we think about birds and their plight in today’s world: marked for extinction on one hand and highly admired as pets on the other; damaging fieldwork on one hand and the aims of conservation on the other. It highlights some common and distressing examples of poor welfare in birds. It also offers some solutions involving simple legislative changes and ways to eliminate some unacceptably low ethical standards in the handling and management of birds.

**Abstract:**

This paper discusses paradoxes in our relationship to and treatment of birds in captive and conservation contexts. The paper identifies modern and new challenges that arise from declining bird numbers worldwide. Such challenges have partly changed zoos into providers of insurance populations specifically for species at risk of extinction. They have also accelerated fieldwork projects, but by using advanced technological tools and in increasing numbers, contradictorily, they may cause serious harm to the very birds studied for conservation purposes. In practice, very few avian species have any notable protection or guarantee of good treatment. The paper first deals with shortcomings of identifying problematic avian behavior in captive birds. It then brings together specific cases of field studies and captive breeding for conservation in which major welfare deficits are identified. Indeed, the paper argues that avian welfare is now an urgent task. This is not just because of declining bird numbers but because of investment in new technologies in field studies that may have introduced additional stressors and put at risk bird survival. While the paper documents a substantial number of peer-reviewed papers criticizing practices counter to modern welfare standards, they have by and large not led to changes in some practices. Some solutions are suggested that could be readily implemented and, to my knowledge, have never been considered under a welfare model before.

## 1. Introduction

It is broadly recognized that birds in the wild need our protection now more than ever because of their sharp decline worldwide. In a report of 2019, a biodiversity crisis in North America was revealed, namely, showing the cumulative loss of nearly 3 billion birds across the avifauna [1]. Spot tests in Australia have shown that woodland bird numbers have declined by over 40% just in the last 20 years [2]. Even avian species that still occur in good numbers show signs of loss of body condition and weight (between 14–28% in a 50-year period), as found in migratory songbirds [3]. Some 30% of all psittacines are now on the endangered list [4,5]. The decline of species, as is well recognized, is chiefly a consequence of human activity [2,3,4,5].

In response to such decline of species and of numbers of birds overall, organizations have been established to help birds recover and survive in their natural environment. The reason why zoos will be discussed first in this paper is that many zoos have now changed their focus away from entertainment and education to involvement in conservation of species at risk. While these countermeasures and the substantial efforts to save species have been remarkable and, at times, spectacular, there is a dark side to these latest developments. First, there are now more birds in captivity than ever before [6], be this as pets, in zoos, or other facilities [7]. Birds have also been captured from the wild, often by poaching and then sold [8,9,10] or captured to participate in conservation breeding programs in zoos. Additionally, in their natural environment, they are handled more than ever before, some with invasive procedures, resulting in suffering and even further decline, contradictorily under the banner of conservation. Welfare standards are either insufficient, not yet formulated, or entirely overlooked. This paper suggests that this is no longer acceptable and needs to change at several layers of legislation, in welfare regulations of journals and institutions.

Scientific information from the field often derives from assessing and recording biological or medical information based on a number of minor procedures. One common one is taking blood samples, which requires a good deal of experience to avoid risk of dehydration and heat or cold stress. When done professionally, it may have little effect on performance of the birds immediately thereafter. However, even procedures usually referred to as minor or harmless, and indeed they are in a medical sense, can have substantial effects on the wellbeing and overall performance and health of an individual bird, be this short-term or long-term. Long-term damage to health and survival is studied less often than short-term damage and, therefore, the reported effects of a procedure can be seriously biased. For example, a rare long-term study found that blood sampling, doing no harm in the short term, reduced annual survival in the first year after sampling [11]. Short-term investigations into the effects of certain procedures may thus be misleading in terms of overall/long-term outcomes [11,12]. Then there are other invasive actions, such as insertion of implants, collection of tissue samples, food samples, force feeding, cloacal lavage, plumage manipulations, and other surgical interventions, sometimes with agreed endpoint of death but also for the sake of evaluating reproductive status. Capture methods in the field include a variety of traps (mist nets, canon and rocket nets), funnel traps, or specific traps at nest sites. Moreover, in order to continue observing birds in the wild, captured individuals tend to be marked in some way, be this with one or several of the various leg bands (color or metal), leg tags, radio or satellite transmitters, wing markings, neck collars, nasal discs, and saddles (the latter two for waterfowl), dyes, or ultraviolet markers [13].

The best and most thoughtful guide to the use of wild birds in research that I have read was that issued by the Ornithological Council of Washington, D.C., USA, in its third edition of 2010 [13]. It is an insightful and collaborative effort that included Laboratory Welfare, Ornithology Organizations, Zoo and Aquarium alliances, various bander associations, and other relevant organizations. Its brief was to consider the impact of any aspect of research on wild birds, including procedures that can stand up to scientific and, thus, ethical scrutiny.

However, the question is whether welfare standards (for birds) have kept pace with the new and increased type of human interventions in the last decades, despite concerned Letters to the Editor in Nature in 2007, stressing the need to include details of welfare information and the three Rs in the method section of a research paper submission [14,15]. Theirs was an important step to voice publicly that many journals did not have an explicit policy on animal welfare [16].

Further, one needs to question whether the principle of the three Rs (Replacement, Refinement, and Reduction in birds or of techniques used) is considered in all field studies that require some procedure as well as in so-called routine procedures used for birds in captivity. There is evidence that welfare standards in some avian studies have been left far behind and have negative outcomes. As many as 50% of birds die within a year of being acquired by zoos [17] and many birds in zoos and in the field are handled in ways that have been shown to do them short-term or serious long-term harm (discussed later). Even for those circumstances that are included in welfare regulations, protection and wellbeing for the birds are not always guaranteed.

Until recently, welfare in birds, other than farmed birds, has not always had the same attention as has rodent and primate welfare [18,19]. Animal welfare research remains biased toward mammalian species within a wide range of facilities (zoological facilities, laboratories, companion animal studies) and welfare of birds in farming contexts. Indeed, the welfare net for birds is so broadly meshed that probably most extant birds miss out on any minimal, considered, or regulated welfare. In zoos, birds may make up to 30% of all exhibits, but avian-focused studies account for less than 10% of all welfare research in zoos in the last 10 years, and then often not involving the same criteria or applied at the same depth of study as in mammalian species [20].

It needs to be stressed that this paper is not aimed at covering the body of excellent welfare work that has been done on the welfare of birds kept for farming purposes (particularly the research on domestic chickens used in the meat and egg industries). In the context of agriculture and research institutes, this work, conducted over decades, has had an important impact, and substantially influenced welfare regulations. It is also stressed that the arguments presented below do not constitute a review but are brought together under a specific separate category called ‘Viewpoint’, which permits one to make a case for a very specific issue. Hence, the paper discusses and cites work directly relevant to the welfare of birds in research contexts and in captivity in zoos. In so doing, it presents results of excellent and detailed scientific studies that clearly demonstrate harm done to birds.

The studies brought together here demonstrate that, despite ample scientific evidence showing a need to implement or improve welfare practices in field research and zoos, these findings often have had negligible impact so far. This is not to diminish the successes and hard work of countless volunteers and scientists who have committed to saving endangered species. On the contrary, the evidence provided here is aimed at strengthening conservation efforts both in in situ and ex situ.

This paper invites debate on how best to solve problems concerning some methods used to gain data or how to prepare for rehabilitation in a way that may not carry high costs for individual birds. It explores alternatives and suggests several solutions at the legislative and duty-of-care levels for new welfare standards. Alternative ways of gaining important data are also proposed. Finally, the paper questions evidence obtained by research that uses flawed or problematic methods in field or rehabilitation work. This paper argues for improved methodologies and explicit welfare regulations in some in situ and ex situ conservation contexts. Ultimately, the paper argues for more rigorous science and improvements in bird welfare in such specific contexts.

## 2. Birds in Captive Environments: Identifying or Avoiding Behavioral Problems

Many behavioral and even physical problems in captive birds are not recognized or regarded as important. In this section it is argued that micro-signals may be crucial in identifying distress or pain. Poor welfare is and remains an urgent area of concern, evidenced by the very high death rate of birds within the first year of zoo ownership and the figure is likely even higher in private facilities [17].

### 2.1. General Behavioral Problems and Their Causes

Birds are usually not classified as companion animals, as are canines, felines, and equines [21]. Seibert [22] made the important observation that birds, especially parrots (among the most favored pet and display species)*,* do not have the same extensive history of domestication as do other companion animals. Quite often, captive psittacine species are only a few generations removed from the wild, and some of them are wild caught. Some species are difficult to breed in captivity or, at least, they do not breed at the rate that occurs in the wild. Dickens and Bentley [23] showed in wild-caught starlings, *Sturnus vulgaris*, that those were housed in outside aviaries bred at normal times, whereas those housed in indoor aviaries did not breed at all [23]. Similarly, starlings were investigated by Bateson and Matheson [24] who showed convincingly that starlings housed in barren cages explored less and showed less confidence than did starlings housed in enriched cages. A study of social isolation in starlings found that such isolation has a negative effect also on the birds’ ability to deal with novelty [25]. Pet birds, even if loved by a human family, may suffer from species isolation, in some cases heightened by a past in the wild [22]. A recent study of African grey parrots, *Psittacus erithacus erithacus* [26], found that social isolation alone shortens the life expectancy as measurable by the length of telomeres [26]. Indeed, birds in captivity often suffer and do so over years, be this because of boredom, loneliness, stress, lack of ability to move, or even as a result of having been given the incorrect or nutritiously deficient food [27]. Examples of typical problems are summarized in Table 1.

In zoos, private or state, and research facilities, there is ample opportunity to improve the welfare of birds. Some facilities may already be near optimal levels of welfare. However, an experienced former zoo Chief Executive Officer claimed in an interview in 2016 that nine out of ten zoos ‘failed’ welfare standards [63] across all species; but for birds the tally may even be worse. At least, a good range of behavioral and welfare problems specific to birds have been identified, as Table 1 shows. Theoretically, having such information should enable removal of many causes of health and welfare problems but this has not been the case so far, at least not apart from some of the most progressive zoos.

Signs of ill-health and psychological problems may be subtle or stereotyped and obvious. The point here is that physical and behavioral problems are highly prevalent in captive birds, be they songbirds, non-songbirds, parrots, waders, or shorebirds, notwithstanding some excellent species-specific research on how to prevent or reduce abnormal behavior [64].

### 2.2. Pain and Emotions

Welfare indicators are, or used to be, chiefly expressed in negative freedoms [65,66,67,68,69]: being free from hunger, thirst, fear, and pain. To be free from these criteria is not as easy to prove, let alone observe, in birds because all four states can occur without visible symptoms. A bird that is bullied or harassed by others may well not be allowed near food trays or a water source. Regular weighing and checking for hydration level can give a reliable measure of food and water intake. This can be important for detection of all kinds of health problems [70,71] but, for reasons of limited resources in time and staff, such checks rarely happen on a regular basis. Weighing birds in aviaries does not necessarily require handling when a simple hidden weighing platform is installed that can be turned on remotely.

Fear can sometimes be gauged by noting alarm calls or hiding behavior but not always. Detecting pain by sight alone is even more difficult to ascertain. Compared to mammals, gauging pain in birds can indeed be difficult. Even with severe injuries or pain-causing internal disease, any typical indices of suffering may be absent [72,73]. Birds in pain may occasionally show eye-lid flutter or eye-closing but not express trembling, screaming, or moaning. ‘Pain masking’ (hiding pain) is a strategy used by birds to avoid drawing any attention to themselves from potential predators [74,75]. While an important adaptation in birds, discovering whether pain is absent or present, is thus not a simple matter.

When humans deal with birds and do not fully understand that the typical signs of pain may not present or be known as they may be in mammals, the outcomes can be horrific. The most scandalous recent failings of welfare in birds were detected in a research project (allegedly for conservation) and first made public in *Science Magazine* in 2017 [76]. A veterinarian levied his objections against a research project on the grounds of the treatment of birds in several experiments, saying that birds experienced “Unrelieved suffering and trauma for experiments that lack real world applicability to veterinary and conservation issues”. I quote and partly paraphrase (to omit names and gender) from the *Science Magazine*:

“The experiments involved captures of wild flocking songbirds, confining them to an artificial laboratory setting singly or in pairs. To induce (further) stress, the experimenter yelled at them, rattled the cages, and rolled the cages swiftly back and forth to prevent perching. In one experiment, birds held captive were so distressed they lost 11% of their body weight within 5 days. In a wounding experiment, although the experimenter used anesthesia before inflicting the injury, no pain relief was provided, which meant the birds woke up in pain from the wounds. In oil feeding experiments, crude oil was fed to one group of birds while a second/control group was not. When the two groups of birds were compared, both had been under so much stress and pain that they experienced the same rate of weight loss. Moreover, the experiments used sparrows. There is little correlation between sparrows and aquatic birds, the species generally affected by oil spills, and studies of penguins and ducks have produced widely varying results. The birds in those experiments underwent prolonged captivity and repeated painful injections and stressful anesthetic episodes before they were killed”.

[76]

Corticosterone levels, detectible in droppings or in feathers, can readily identify stress levels in a bird but to detect depression (as a chronic condition) by sight alone takes substantial experience and careful observation of micro-signals. Avian micro-signals have been studied only rather recently but are beginning to show their relative importance in physical and psychological health assessments in birds [77,78,79,80]. The first insight needs to be that birds experience pain, suffering, and emotions of considerable complexity [62]. The second is that individual birds even of the same species may react very differently to the world and will do so according to individual personality traits [81,82]. There is now plenty of evidence that personality or temperament has a good deal to do with a bird’s reproductive success and overall health [60,61,83,84]. They can suffer ‘mental’ health issues of varying degrees of severity—the worst of which, in cockatoos (family Cacatuidae), are stages of catatonic immobility and swaying while not attending to any external stimuli [27]. They may also perform self-mutilation—not just pulling out feathers but biting off a toe or otherwise inflicting self-harm [22,85,86].

Current research findings have long since outgrown the opinion Weary and Fraser expressed in 1995 [87] that an animal’s fitness needs are met by providing adequate food and shelter. We now know that it is not that simple. The discovery of avian cognitive abilities [88,89], personalities [82], and emotional complexities [74] has certainly widened the areas of welfare concerns.

Complex emotions in birds are not just inferred. In the last two decades they have been confirmed in two ways: one by studies of the brain, another by study of hormones and neurotransmitters. The hormones circulating in bird brains are much the same as in mammals, even including prolactin (once thought to be a uniquely mammalian attribute) but now known to have multiple functions in avian reproduction [90,91,92]). Then, there are also neurotransmitters in birds as in mammals, such as serotonin [93,94] and dopamine [95,96]. Serotonin (5-hydroxy-tryptamine) is a monoamine neurotransmitter that controls mood, including alleviation of stress and promoting relaxation. Dopamine (3,4-dihydroxy-phenethylamine), partly with its own network, is commonly described as the ‘reward’ neurotransmitter. It plays important roles in executive functions, motor control, motivation, arousal, memory, and reward [97,98,99].

Given this arsenal of neurotransmitters in the brain and circulating in the body, emotional complexity in birds is a fact rather than a speculative or anthropomorphizing assumption and needs to be considered in welfare.

As in mammals, birds have the means to control some if not all emotions via lateralization of brain function. The left hemisphere also discriminates objects (food from non-food), whereas the right hemisphere is responsible for the expression of intense emotions (such as fear or aggression) [100]. Intense emotions are largely controlled by the posterior and medial archistriatum, the avian homolog of the amygdaloid complex in mammals [101] that was found to be involved in the control of social behavior through its influence on the affective state [102].

Fear is a powerful emotion and, while it can have some life-saving function in the short-term, prolonged fear is very harmful for any of the vertebrates. Years of painstaking research by neuroscientists, endocrinologists, biologists, ethologists, and cognate disciplines have shown that fear is a powerful agent also in birds, with a range of ramifications for basic health and reproduction. Agnvall and colleagues [103], for instance, found in junglefowl (*Gallus gallus*), that, in cases of low fear, basal metabolic rates are higher, feeding efficiency is greater, plasma levels of serotonin are higher, and exploratory behavior is more common compared to birds with high levels of fear [103].

**Figure 1:**
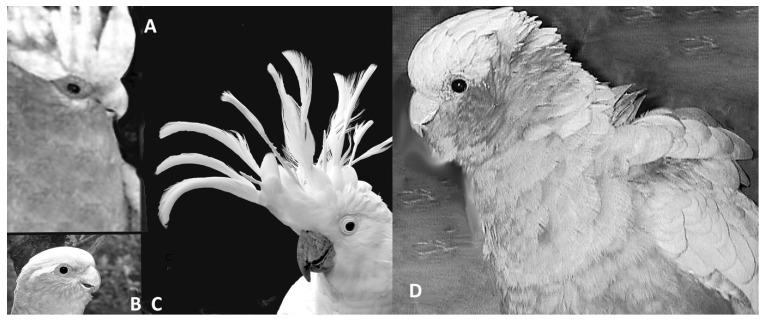
Expressing emotions via vocalizations, body posture, but also via feather positions. Among the easiest to ’read’ are the signals given by birds with crests. Crests can signal threat displays, curiosity, interest, play readiness, alarm, and many other emotions. All cockatoos have crests, including galahs. (**A**) A play face by an adult galah: wings out slightly and crest raised to full height. (**B**) Adult galah without crest erection, neutral but alert expression. (**C**) Adult sulphur-crested cockatoo alert but no sign of any animosity and ready to play or communicate. (**D**) Angry galah, back of the neck feather raising is common among many species but the addition of raising of breast feathers makes it abundantly clear to other galahs not to step any closer. Note that (**A**,**B**,**D**) are images taken by the author of the same bird in the same year. (Photo credit: G. Kaplan).

In other words, for the first time we know that birds have the same complement of hormones and neurotransmitters (or their equivalents) as mammals, and this indicates that birds may have the same or similar regulatory processes for emotions. While the right hemisphere in birds and mammals is involved in expression of intense emotions, the left hemisphere may inhibit some of these strong responses [104,105] provided the individual is not overwhelmed. These findings are clearly of relevance to welfare in birds [52]. Understanding avian emotions can be achieved by systematic study of certain changes in their postures, feather positions, or vocalizations and observation of behavior. Birds have open mouth displays or, rather, open beak displays, which may have a number of signal functions. One of them, together with other body signals, can indicate fear [105]. As a threat, a number of bird species open their beaks, even without vocalization but sometimes associated with hissing or breathing sounds as in geese [106]. Galahs, *Eolophus roseicapilla*, and sulphur-crested cockatoos, *Cacatua galerita*, also use hissing sounds together with open beaks, very similar in sound and appearance to the same display as in snakes, lizards, and some owl species. In galahs and all crested cockatoos, raising of the crest may not just be in alarm but can occur in states of friendly arousal, in play readiness, and in affiliative gestures. In cockatoos, there may be dedicated positions of crest raising for the expression of very different moods. The feathers that flank the beak (ear coverts) can be ruffled to express anger and possible readiness for attack (Figure 1). Lowering or flattening of feathers is usually associated with fear, but this commonly involves the whole body rather than just the head [107].

Feathers play an important role in thermoregulation [108]. Beyond physiological functions, feather positions (sleek, erect), which can occur on most parts of the body, and in parrots specifically in the head and neck region, have long been known to have signal function [109]. Fluffing feathers below the beak (readily observable in many parrots [110]) can be an expression of a relaxed or even satisfied state. For close, conspecific interactions, these facial expressions are powerful signals emitted with minimum energy expenditure. In most cases, such signals are effective only in intimate situations. In addition, like mammals, birds have a wide range of body postures. Such signals (some are referred to as micro-signals) have not been studied or used sufficiently to identify states of emotions in captive birds [30,79].

### 2.3. Enrichment

Enrichment is the alteration of the animal’s environment or activities that can be shown to have some beneficial effect on the animal in question [111]. Enrichment has been regarded as one effective way to improve the welfare of birds. While the term ‘enrichment’ appears unambiguous enough, it has often been misused or misunderstood at the operational level [22]. Errors in providing enrichment can be due to anthropomorphizing (thinking what is good for humans ‘must’ be good for animals) or to thinking that any addition of any kind to the environment or timetable of a captive animal is *ab ovo* ‘enrichment’. This is not so.

As many studies have now shown, welfare improvements usually lead to more naturalistic behaviors, mimicking or encouraging some small aspect of the animal’s natural repertoire [31]. Scientific literature on animal welfare has translated ‘beneficial’ into measurable categories of behavior and physiological responses and these have been taken as a guide to what constitutes enrichment. Welfare initiatives, also for birds, today generally fall into several categories usually referred as “food-based” [31] “structural” [18], “sensory” [112,113,114,115] “environmental” [35,116,117], “social” [25,30], and “cognitive” enrichment [32,118,119]. Each category can be tested separately but, ultimately, all of these categories are relevant to each and every bird. Providing a perfect environment for birds is not one in which everything is ready-made but in which birds can express natural foraging and nest-building behavior, for instance, or in which they may need specific skills to solve a problem [120].

Some zoos have taken a series of important steps to maintain and/or develop a comprehensive and sophisticated welfare program in line with concerns expressed on how avian welfare is being handled [121,122,123,124]. However, the requirement to implement such a plan may not always be matched by any form of oversight to ensure that even minimal standards are met. [63,125].

**Figure 2:**
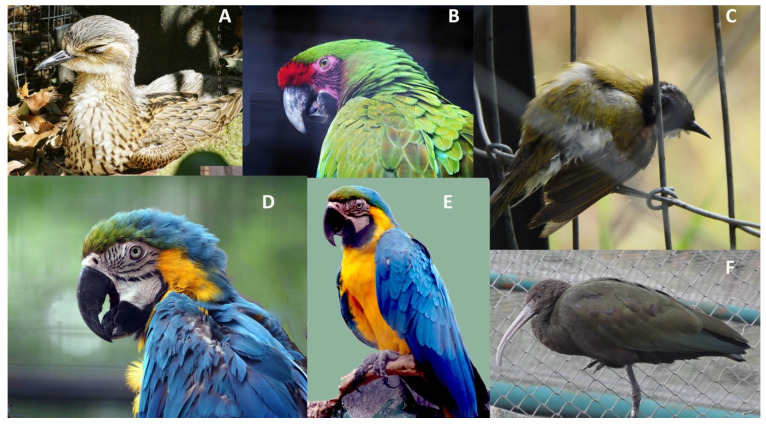
Overt signs of discomfort or distress. From left to right, top row: (**A**) Australian bush stone-curlew, *Burhinus grallarius*, a very shy bird, shutting its eyes when watched by humans and not being able to get away. The eye closing, in the case of largely nocturnal birds, may simply be a sign of brief sleeping bouts. In other cases, it can be a sign of stress, pain, or both. Closed or semi-closed eyes in diurnal birds can indicate acute cases of illness or stress. (**B**) Military macaw, *Ara militaris*, turning away and raising feathers on the nape of the neck is a passive–aggressive posture. (**C**) Small songbird in active-aggressive mode in fear and about to flee: drooping flight-feathers, arching back, and raising feathers on the back. Bottom row: (**D**,**E**) Blue and yellow macaw, *Ara ararauna*. Two different postures indicating deep distress, first image: wings raised and slightly fluffed and head slightly forward; second image: wings hanging and head slightly to one side and lowered. (**F**) Glossy ibis, *Plegadis falcinellus*, long neck retracted and head slightly to the side, standing on one leg. The raised leg may well indicate a brief resting respite for the foot, without problems, but in some cases may suggest problems with a foot (injury or swelling) and as shown here, it is likely that this bird is in pain (after watching it for several hours, it never changed legs and kept just the left leg tucked away). Another variant is to adopt a sleeping position with the head buried amidst flight feathers. However, this posture, often with eyes shut completely, hints at more than psychological distress and rather suggests some form of illness (Photo credit: G. Kaplan).

There are ways of assessing the well-being of a bird from a distance, simply by body posture and eyelid and feather positions. Admittedly, these signs are indeed often minimal, especially when a bird is injured and in pain, as outlined above. A few examples of micro- or postural signals that tend to indicate some physical or mental distress are provided in Figure 2 (photos taken in state-run zoos). Minor variations in body posture or squinting of eyes may well indicate a range of negative states such as stress, discomfort, and depression. While research has begun to identify the importance of the many micro-signals as health and mood indicators, it is quite possible that these insights are as yet not widely shared among zoo or field-staff or their importance remains doubted and dismissed as subjective or even ridiculed.

A recent paper made the important proposal that diversity of behavioral repertoire may be an indicator of positive welfare [126]. This is a very useful suggestion, indeed, because one common element in the behavior of the birds shown in Figure 2 is that they barely moved or even changed posture. Hence, a lack of varied activity or any activity at all, particularly in social species, may well indicate that a problem exists. However, this requires more than a minute of observation.

#### 2.3.1. Physical Environment

It has become clear from the research conducted so far that, sometimes, the difference between a problematic and an appropriate cage environment may appear very minor to the human carer. One such variable concerns perches, studied in detail in poultry housing but not in other avian species [127].

For instance, the feet of most raptors are not designed for long periods of resting on the same type of perch. However, if they have no choice in captive environments, they tend to develop foot injuries such as ‘bumblefoot’ (*plantar pododermatitis*), an infection of staphylococcus bacteria, turning into a substantial and painful abscess of the foot (toes or pads), which can become very severe, lead to substantial swelling and even lameness and is associated with difficulties in walking and perching [128].

Early fieldwork showed very clearly what type of perching birds prefer, at least in raptors [129]. Size variations, texture, and spacing matter a great deal: Too many perches can obstruct flight, too few can prevent flight and wrongly sized, slippery, or contaminated perches can aid the development of infections (of feet). For healthy feet, perches should preferably not be smooth but have different textures, as the bark of various species of trees can provide, and they should also be angled in different ways to ensure ongoing exercise of feet and legs. Importantly, lack of flight leads to weakness of flight muscles and decline of general overall health.

Placement of perches should also always include perches as high up within the enclosure as possible without forcing the bird to crouch. Psychological health may be compromised and activity patterns change or cease, if a bird cannot perch [130] or hide from something that is perceived by the bird as a risk or danger (and humans are chief candidates for being perceived as danger). The matter of location of cage and position of perches may appear a negligible matter but it is often pivotal for a bird’s health. Indeed, it is one that might well also affect the health of millions of pet birds [6,130,131,132].

#### 2.3.2. Multispecies Housing

Multispecies housing of birds, particularly in zoos but also in sanctuaries and pet shops, creates a range of challenges that are often not identified. It would seem that few zoos, perhaps 10% of the roughly 10,000 zoos worldwide and of zoo designers [133], have the expertise to realize the complexity of desirable conditions in multispecies housing [134]. Rarely is compatibility checked in detail and this can affect the bird’s physical and psychological health, as well as its breeding performance [135].

In some zoos, birds from different continents and vastly different habitats and lifestyles are put together, i.e., not to create a window into an ecological niche or give visitors an understanding of actual multispecies cohabitation, as some leading zoos now do (creating avian conservatories), but to make it more attractive for the public. In one advertised aviary, zebra finches were placed together with macaws and galahs with songbirds from Europe and African grey parrots with starlings (birds from four continents). It may be a nice experience for zoo visitors to step into a walk-through aviary. However, the question has been asked whether the welfare of birds has been considered since they cannot escape. It seems that the larger the aviary and the better the flight spaces, high roosting spaces, and foliage cover are, the smaller the negative effect on birds are [136,137].

The susceptibility to poor health outcomes because of multi-species housing varies within species and between species (1) at different times of year (in and out of breeding season), (2) in different ecological niches, (3) according to foraging contexts, and (4) according to personalities. In the context of captivity, the most obvious interspecific observable conflict is one bird being chased by another. Such animosities can prevent proper feeding in either of the birds (chased and chaser). Multi-breeding pairs of the same species can also be geared to intraspecific competition. Persistent stress is just one among the many risks of multiple species housing [138].

‘Compatibility’ is not just of concern in mixed species enclosures but is also an issue for same-species placements, possibly even from similar geographies and climate zones. Compatibility is a complex area and final selections would need to be carefully researched and continuously monitored once the species have been housed together [134,135]. Even species of similar size and from the same ecological niche require a careful behavioral assessment to avoid competition, aggression in perpetrators, or severe stress in the victims. For example, a nest site study of Gouldian finches, *Erythrura gouldiae*, and long-tailed finches, *Poephila acuticauda*, showed convincingly that Gouldian finch pairs are aggressive towards each other (intraspecific competition) whereas long-tailed finch pairs are not, but they are very aggressive towards Gouldian finch pairs [139]. The result is that Gouldian finches are more likely to be losers in competition for nest sites against this closely related species. Hence, it would not be advisable to place these two species into one aviary because such interspecific competition and aggression can place the losers under considerable stress, crucially because they cannot vacate the area, as they often would in the natural environment [139].

In many species, such as cockatiels, *Nymphicus hollandicus*, and others [140,141], individual birds might even fight with or try to avoid each other. Some might prefer to switch partners [142,143] but others would prefer to go to a different neighborhood in their natural habitat. If they had been paired up artificially, they may perform poorly in reproduction [144].

Some avian species are territorial with different requirements than others that are non-territorial. Matters of space in territorial species tend to be different than in colonially breeding species [145]. Territorial birds, in general, assess risk and choose safe nesting sites and try to protect themselves against potentially dangerous neighbors [143]. Even if it is well researched which species are compatible and which are not, micro-social environments may alter social relations between members, even between the same species, let alone between different species. Competition for space, food, and flight space can cause tensions, aggression, and often distress, with a measurable effect on stress hormone receptors [144]. Stocking density alone can adversely affect reproductive success [145]. Recent studies have recognized that the pragmatic elements of multispecies housing (better use of space and adding interest) have to be matched by careful consideration of potential risks and stresses [146].

Most abnormal behavior is actually predictable. An African bird exhibit in one major European zoo may serve as an example. The mixed-species exhibit of African waterbirds included several species of flamingos, spoonbills, a variety of storks including marabou storks, *Leptoptilus crumeniferus*, and pelicans. One would not expect any breeding activity, let alone breeding success in that display area for one crucial reason: marabous and pelicans steal eggs and also consume relatively large live nestlings of other avian species. A number of pelican species are even cannibalistic, i.e., feed on the nestlings of their conspecific neighbors [147].

Marabous are indiscriminate feeders, omnivorous in the widest sense, be it spoiled human food or live nestlings. Hence, flamingos and spoon bills would not stand a chance in defending their brood, and so will not try to breed. The zoo finally built a new enclosure and, crucially, they removed the marabou storks (but not the pelicans). Still, having made some changes based on behavior and needs of the waterbirds, the birds are now healthier and even breed [148]. The question is: why were these facts about the predatory foraging behavior of marabou and pelicans not checked before the birds were placed into the same aviary with defenseless waterbirds? Nest predation risks have been researched for a considerable time and serious threats, such as the ones those two species represent, are known to affect daily behavior even outside the breeding season [149].

Many errors, as described above, are fundamental errors. They may reflect the fact that too little expertise in animal/bird behavior is used, and the literature is either not seen as relevant and thus ignored or not even known. Research shows clearly that knowing the behavioral profile of a species, its typical environment, and behavior patterns generally is a vital precondition for good welfare and indispensable for birds raised for release.

At the opposite end of the scale of housing is total species’ isolation. Some individual birds, usually large “showy” parrots such as macaws and sulphur-crested cockatoos, have at times a different problem: They get no exposure to other birds of their own or compatible species. They are on their own. They may be wing-clipped and placed to sit alone on a perch near the entrance of zoos, as a kind of exotic invitation for zoo visitors. It is difficult to imagine anything more inappropriate at the physiological and psychological levels. They may have no mobility or no conspecific social company and, for species that roost and travel in pairs or family groups, they miss out on preening or affiliative gestures, enrichment, or distraction to the detriment of their own health and well-being [150].

#### 2.3.3. Problematic Medical Interventions

Some of these social isolates and birds in open flight cages are deflighted or chained. It is usually the former. Surgical procedures of limiting or preventing flight, be this via pinioning (amputation of the wing tip) or tenectomy of the supracoracoideus muscle have been performed and some techniques have been discussed as to which one may be more effective [151] but not whether they were defensible from the point of view of welfare. Some have claimed that there is no evidence to support the claim of harm being done [152,153] but very few studies have been conducted and evaluate any potential long-term effects of deflighting. Among the few are studies on deflighting in flamingos, a popular zoo species because of their attractive display characteristics [152,153,154]. Flamingos are predominantly ground-walking and ground-foraging birds. Choosing deflighted flamingos thus seems a strange choice as a research subject for testing the effects of removing part of the wing to prevent flight. Flamingos tend to fly only when relocating to another salt-lake. Their daily routine is barely disturbed by limited use of wings (provided pain thresholds are low). Flamingos are, thus, not the best species to prove that pinioning is a benign procedure. Another study examined whether flight restraint raised corticosterone levels in flamingos. Results showed that the level of corticosterone was not affected significantly; but the researchers admitted that flamingos are ‘reluctant flyers’ and usually walk [155].

Some improvements have occurred in as far as some surgical interventions common in captive zoo birds have gradually been abolished, but not all and not everywhere. Standard/common procedures in zoos include surgical removal of spurs, of anterior toenail in ratites, salpingectomy in parrots, devocalizing birds, cauterizing feather follicles to eliminate flight potential, and, perhaps worst of all, surgically ‘modifying’ the beak; one method is called ‘disarming’ done in parrots, which may include trimming the beak or nails or even splitting the beak to control mate aggression. Finally, pinioning of waterfowl (amputating the last section of the wing) to prevent flight is a permanent surgical alteration of the individual bird and, as in other procedures, can result in chronic pain. The important point that Klausen made is that none of the procedures used was based on scientific research or medical grounds but purely on practicalities and traditions [148], have little to do with welfare, and, indeed, may be counter to any minimal welfare standards.

The idea of ‘happy’ and well-adjusted individual birds in captivity is a long way off and, depending on context, perhaps not an entirely realistic goal. In 2011, Leus and colleagues [17] published a paper on the sustainability of avian and mammalian zoo populations just within the European Association of Zoos and Aquaria (EAZA), showing distressing results for birds, as mentioned before: 21% of bird acquisitions die within a month of zoo ownership, another 32% within a year, and only 20% of birds within the zoo’s studbooks were breeding. The record might have improved over the last decade, but it is worth remembering that these are basic life/death data that can be taken from general statistics [17]. Such data do not come near questions of overall welfare, let alone well-being [156,157].

#### 2.3.4. Veterinary Response to Interventionist Practices

Some years ago, the American Association of Avian Veterinarians (AAV) made the following position statement.

The AAV does not support any surgical procedure that permanently and irrevocably alters avian anatomic structure or function, with the following exceptions.

(1) The procedure(s) is deemed necessary for the safety, health, husbandry, and well-being of the bird(s) and cannot be accomplished by other nonsurgical means such as an avicultural husbandry management practice;
(2) The procedure(s) is humanely performed in a valid research setting where such a procedure(s) has been approved by an institutional animal care and use committee or an appropriate oversight organization that considers the procedure(s) necessary for the study; and
(3) The procedure(s) is deemed necessary by the administering veterinarian and is not on the list of condoned procedures in this statement [158].

This position statement has had considerable influence and (some) zoos have realized that pinioning (and hopefully some of the other surgical interventions) must cease and be replaced by evidence-based practices.

## 3. In Situ and Ex Situ Conservation

The second part of this paper will concentrate on some of the pitfalls of conservation practices both in zoo conservation breeding facilities and in field studies. Aspects of these new practices will be highlighted, which, in many ways, have so far escaped scrutiny or failed to be guided by acceptable welfare standards. After critical assessment of the literature of mammalian and avian research papers, it is proposed here that a raft of new welfare tools will be needed to cover captive breeding programs and some field practices.

As is well known, the understanding of the role of zoos has shifted substantially over the years. From having served as educational or entertainment venues for the public, some leading zoos and botanical gardens are now heavily involved in breeding species that are in the endangered or highly endangered categories [159,160]. One goal was to create ‘insurance populations’ as a ‘backup’ should a species become extinct in the wild. In 2002, the International Union for Conservation of Nature (IUCN) published its ‘Technical Guidelines for ex-situ Conservation’ and from there on a distinction was made between in situ and ex situ conservation [161,162].

In situ conservation refers to boosting species numbers within the natural environment. In the past, this may have been achieved by declaring national park or sanctuary status to areas in which vulnerable species occurred, increasing protection and strengthening legislature against trade or poaching. In such in situ attempts, additional work may have also involved removal of introduced predators, provision of nest boxes, and additional planting of relevant flora for the species concerned. However, these examples of minimal interference are becoming increasingly rare. Successes have resulted mainly from targeted protection. For instance, Lear’s macaw, *Anodorhynchus leari*, changed from Critically Endangered to Endangered as a result of active protection of the Toca Velha/Serra Branca cliffs in Brazil and, at the time, also from enforcement of legislation (such as hunting bans) and harvest management measures [163]. Another is the case of bolstering numbers and reintroducing scarlet macaws, *Ara macao*, in Los Tuxtlas, Veracruz, Mexico [164], or the case of conservation activities in Costa Rica, where local success in stabilizing numbers of scarlet macaws included the creation of a local conservation organization to coordinate environmental education, artificial nest construction, and networking among stakeholders and with governmental authorities [165]. Such optimism may have worked at the local level some 15 years ago but since then deforestations and transformation for grasslands into farmland, for housing or other reasons, has increased and has led to substantial transformations of landscapes [166].

Any simple calculation can tell us that, no matter how strong the desire may be to restore nature and species to their former glory, there is no corresponding expansion of protected areas relative to ever-increasing human expansion. Indeed, the number of suitable habitat patches is decreasing rapidly for most species, including birds. There is an illusory aspect to returning animals back into the wild, as Braverman’s paper in 2014 titled ‘Conservation without nature: the trouble with in situ versus ex situ conservation’ so well illustrates [167]. When birds have declined or gone extinct in one area, there are reasons for their disappearance or decline and some of these reasons may not fall into a category that society or individuals can or want to change (expansion of population and industries) [168].

To keep up the idea that all we need to do is breed up animals in numbers and then release the captively bred populations back into the wild, as Bravermann argued, “requires the construction” of a nature ‘out there’, that actually no longer exists” [167]. Indeed, in situ conservation is now often reduced to small band-aid measures restricted to reserves or remnant forest and grassland areas. While augmentations (increasing numbers of vulnerable species in one area) were relatively successful, reintroductions and translocations were generally not, especially if they were sourced from captive populations (raised in zoos). In situ efforts are usually not funded by government sources and, with a few notable exceptions, tend to depend on donations and on the devotion of a band of dedicated volunteers. In situ conservation is, thus, mostly targeted, small-scale, and unfunded.

By contrast, ex situ conservation, largely powered by zoos, has substantially increased. Ex situ refers to captive breeding programs intended to maintain genetic biodiversity and build up numbers in species that are vulnerable or highly endangered in the wild. For instance, it is well recognized that the reintroduction of captively bred Californian condors, *Gymnogyps californianus*, was ultimately successful because of the awareness and inclusion of two important variables: the knowledge of and importance of imprinting and the use of adult condor mentors [169]. Nevertheless, the number of failures has also been substantial. Some of the problematic cases relate to fieldwork methods that may need close attention and action from a welfare point of view, as will be outlined in the next sections.

Ex situ breeding and conservation programs are expensive and often well-funded (be these government funds or donations). Conservation Planning Specialist Groups (CPSG) have been created at the international level, with the explicit aim of involving zoo and aquarium associations, for joint actions between in situ needs and ex situ implementation. There is now also an Integrated Collection Assessment and Planning (ICAP) framework to guide zoos and aquariums on conservation priorities that also provides for in situ field support and seeks integration of in situ and ex situ efforts: That is, achieve collaboration between zoo and aquarium associations and field-based conservationists. [170].

### 3.1. Some General Methods for Field Data

The new focus on conservation and threatened species suggested that more field research was needed. Indeed, as many new data as possible ought to be collected to understand what has made a species decline in the natural environment and how it could possibly be saved. Decades of concerted efforts have gone into answering these questions. As a result, we have fairly accurate data now of most endangered avian species. However, the rush for data may well have contributed to the fast-tracking of methods with negative health consequences for the targeted birds. Field et al. [171] argued recently that the rush for data made too many institutions and individual researchers accept maltreatment of wildlife, if at times grudgingly [171].

Methods demonstrably harmful to birds, even risking their survival, appear to be based on an ethical blind spot about how data can be collected in the field [171]. Worse, some of these methods are condoned by research facilities and have become far too numerous to ignore. Moreover, the problem is not just confined to universities and other organizations but extends to journals publishing the findings. In 2019, Field and colleagues started by looking at 206 relevant research journals and found, to their dismay, that a third of them have no explicit animal welfare policies and, in others, they were weak or incoherent [171]. They were right in pointing out that, if journals outlawed certain methods, researchers would take note of basic welfare principles. They argued, and I quote:

“Sound science requires animal subjects to be physically, physiologically, and behaviorally unharmed. Accordingly, publication of methods that contravenes animal welfare principles risks perpetuating inhumane approaches and bad science”.

[171]

Burden and colleagues had already pleaded in 2015 [172] for ‘better science’ which, in part, could be achieved by the application of basic welfare principles such as the three Rs in wildlife research (Replacement, Refinement, and Reduction), as had occurred for laboratory animals earlier in the USA [124,173], Canada, Australia, New Zealand, and in Europe [174,175].

A year after Burden et al.’s paper [172], Zemanova [176] published a paper bringing together copious examples of available and innovative alternative methods, i.e., showing the principle of the three Rs at work, be this in genetic or behavioral studies. Zemanova demonstrated convincingly that methods are already available that are non-invasive and superior in producing reliable data [176]: For example, instead of capturing birds and taking blood samples (both highly invasive and stressful strategies for mammals and birds alike [177]), various studies have found that excrements [178,179] and feathers [180,181] can produce reliable results without subjecting the bird to invasive procedures [182]. In foxes it was demonstrated that DNA could be extracted from their footprints in the snow and, thus, did not require trapping and handling them [183,184]. Such are or could be important steps for avoiding stress and death of birds. Just being captured, handled, and struggling to get free may lead to an onset of exertional or capture myopathy [185,186] known to kill cranes (immediately or delayed) and many other species during capture and translocation [187,188,189].

Another area, to be discussed below, concerns the study of bird movements, especially migration, secured often by means of attaching short- or long-term apparatus (telemetry) to the body of birds.

While the IUCN (International Union for the Conservation of Nature) continues to affirm that one goal of conservation is the maintenance of existing genetic diversity and viable populations of all taxa in the wild, the threats to biodiversity continue to expand [190]. The IUCN, in fact, admitted in 2002 that it will not be possible to ensure the survival of an increasing number of threatened taxa without effectively using a diverse range of complementary conservation approaches and techniques including, for some taxa, increasing the role of ex situ conservation [161].

Ex situ conservation, now a widely accepted practice, includes a considerable range of activities: the storage of embryos, semen/ovule/DNA; captive breeding through the establishment of field gene banks and livestock parks, with many of them very successful in what they set out to do, particularly in plants and small vertebrates (such as lizards and frogs). However, programs specifically targeting birds have rightly sparked controversy. Of those released, the death rate is too high, nest abandonments too frequent, and breeding success overall lower than that of the wild counterparts. This may be attributable to inexperience and stressful experiences post release. Alternatively, bringing species back from the very brink of extinction has been shown to be often extremely difficult and, at times, impossible. An example was the case of the now-extinct dusky seaside sparrows, *Ammodramus maritimus nigrescens*. There were only 13 individual birds left in the wild and 12 of them were males [191].

Some captive breeding programs, be they turtles, amphibians, or fishes, have become very important conservation tools without excessively draining resources. In birds, it is often far more difficult and may offer few returns for an enormous outlay in cost, energy, and time. In an important critique, Snyder identified a number of limitations [192]. Among these are the high costs and difficulties associated with establishing sustainable captive populations. The breeding program for the Alalā Hawaiian crow, *Corvus hawaiiensis*, for example, cost in excess of $1 million annually, shared by governmental partners (such as the U.S. Fish and Wildlife Service and the State of Hawaii Division of Forestry and Wildlife) and San Diego Zoo Global, as well as numerous foundations and private donors [193,194]. The territorial Hawaiian crow was critically endangered and became extinct in the wild in 2002. When only 12 Hawaiian crows were left in the wild, they were taken into breeding facilities. Eventually, 27 captively raised juveniles were released. Of those 27 birds, 21 birds died and 6 were recaptured and, as far as reported in 2014, they had to be admitted back into captivity [194].

One of the very detailed and informative reports concerned the decline of the greater sage-grouse, *Centrocercus urophasianus*, whose regional populations in Canada and elsewhere in North America have declined by a staggering 98% [195]. A number of agencies suggested to save the species and various methods were examined, such as augmentation, reintroduction, and translocation. The sage grouse has been well studied and its sharp decline was well documented. As in most cases, the reasons for sharp population declines tend to be manifold and the result of multiple confounding changes. In the case of sage grouse, there were identified changes in the environment such as encroachment of plants [196], disease such as the West Nile virus [197], and a variety of industry activities, hunting, and other human undertakings [198,199,200] that made the project largely fail. All known successful translocations have involved at least some birds that have been captured from the wild.

Failures are always distressing also because of the years of work that had gone into saving a species. Presumably, some failures (suffering and death of the released birds) could have been avoided with proper training, understanding of the life history, and behavioral and ecological requirements of the species concerned.

From its very inception, ex situ conservation has had plenty of skeptics, doubting the ability of zoos to successfully deliver the establishment of long-term, self-sustaining insurance populations for a large number of threatened species. Moreover, crucially, it was asked whether such work could achieve the momentous transformation of getting captive populations reinserted into the natural world and whether the efforts could make a measurable difference to the preservation of any threatened species [201].

It has recently been pointed out that even in conservation fieldwork the behavior of birds (ethodiversity) has been a neglected dimension in studies of biodiversity [202]. Curio warned some decades ago that conservation needs ethology and that it will not do well without understanding behavior using appropriate methods of gathering data and coming up with solutions [203]. That advice has been increasingly ignored. At the very least, it means understanding the behavior of birds, understanding the timetable for appropriate behavior to occur in juveniles, and providing opportunities pre-release to express and test them. Every individual raised in captivity should have had exposure to and learned *pre-release* (1) about predators (how to recognize them and how to respond since we have known for some time that predator avoidance is a socially acquired skill [204,205,206], (2) to recognize and find food, where to look and how to process it [207], (3) how to socialize with wild conspecifics [208], (4) to recognize and respond appropriately to calls made by conspecifics and heterospecifics [209], and, in some species, (5) how to build nests [210,211]. Most birds, as juveniles, acquire such skills by observation of parents or wider family groups: These skills need to be learned and competency in all those skills (and confidence) is essential for survival.

To give just two examples of high-priority reintroduction attempts with poor results so far are the Hawaiin crow, already mentioned, and the Australian regent honeyeater, *Anthochaera phrygia* [212]. Both are critically endangered, and both are considered flagship species, i.e., suggesting that their successful reintegration into their natural habitat will benefit many other species as well. Juvenile corvids belong to the most supervised and guarded offspring in the bird world. They learn by watching parents and siblings [213] and, when older but still unbonded, favor staying close to other juveniles in the wild [214,215]. Predictably, if the co-released juveniles are about the same age and equally inexperienced, they would most likely fail in the long or even short term. Similarly, none of the releases of captive-reared grouse, mentioned before, produced viable local populations [195]. In regent honeyeater (in Australia), captively raised birds have so far survived. However, post-release, of 28 nesting attempts by 26 released pairs, only two viable offspring were produced, i.e., over 95% of all nesting attempts failed [216]. Some of these nests were also predated by small native mammals (sugar and greater gliders) [216]. We know that parent-induced stress also changes the feeding behavior of birds [217]. Whichever group of vertebrates is studied, the majority of conservation efforts in mammals and birds relying on captively bred species rarely exceed a 50% mark of success [194,218].

One of the central themes in avian cognitive research is the importance of learning in birds, thus a topic of great relevance to studies on captive breeding and reintroductions. Yet Berger-Tal et al. [219] found in their extensive survey that, in publications on captive breeding studies, learning was only discussed in 1.45% of papers. Furthermore, behavioral issues in reintroductions were mentioned in only 2.15% of the papers, studying wildlife disease management in 0.96% of the papers, a further 2.03% discussed human–wildlife conflict, and a mere 0.2% included vigilance behavior, despite its central importance for predator detection [219]. This suggests that key elements (the actual and predictable behavior of birds) may have been either ignored or not fully integrated in the rearing of birds destined for release. Until there is a revival in linking ethology with reintroductions in captive breeding programs and in behavioral studies of the same species in the wild, the failure rates of sustainable reintroductions may well continue to be high. Of course, this depends on circumstances.

The survey by Berger-Tal et al. [219] should raise the alarm but, generally, does not seem to have done much to zoo practice. A similar lack of appreciation of the complexities of birds’ cognitive and emotional needs and capabilities (underplaying the effects of pain and fear, for instance) can be found in fieldwork, as will be highlighted in the next section.

### 3.2. Conservation and the Demand for Data: The Technological ‘Solution’ (Telemetry)

The last and equally important point of this paper concerns fieldwork of intact or dwindling populations. Birds are now studied in situ in greater numbers than ever before. Declining numbers have invited more comprehensive investigations into the causes of the decline of birds worldwide be this for sea, shore, or land birds. Unfortunately, such legitimate enquiry has resulted in increased and substantial interference with remaining avian populations in the wild and has often done so to their detriment. Migratory, vulnerable, and endangered species may now be the most targeted and handled species in the wild. The interference comes in the form of mist netting and an array of techniques of marking birds with GPS devices together with a range of fittings and harnesses used to provide data on movements of birds, on nesting success, and return rates of birds that migrate, among other things as will be outlined next.

Telemetry is a popular technique for studying dispersal, habitat use, migration routes, and mortality in birds. It involves automatic transmission to receiving equipment for monitoring, indeed a wonderful research tool. Data loggers and such basic equipment were already in use in the 1960s but the number of studies using modern transmitter devices has steadily increased from decade to decade [220]. Modern avian devices are not like PIT Tags (implantable transponders) used for identification of individuals that were generally found to have no direct impact on birds [221] and not like wingtags. They have become more sophisticated even in the last few years and now the smallest tracking devices, often used for tracking cars and children, even have solar power.

Translated to small and lightweight birds, however, these devices are still relatively large external fixtures. While they are scaled according to weight and size of species, GPS backpack devices may range from 10–75 g, patagial mounts from 23–53 g, and avian leg bands from 2–26 g while glue-on devices are generally much lighter, ranging from about 1.3–28 g. How they are affixed is usually by four different methods: glued on (now rather rare), harnessed, stapled, or anchored subcutaneously (usually also with an additional harness). Location of the affixed device is usually in three different locations: on the back, on the wing, or as a leg loop (on one leg) or subcutaneously anchored.

Recent research suggests that leg loops are the least successful (in terms of staying on the subject), while shorter-term studies have used predominantly fixtures on the back. One study used several species of migratory geese and found that those with attached harnesses had lower return rates [222]. Another 18-year-long study of the southern dunlin *Calidris alpina schinzii*, a small migratory wader in Finland, confirmed that the attachment of geolocators resulted in reduced survival [223]. Importantly, death rates increased more markedly the longer the birds carried the device [223].

The measurements derived from any attached GPS devices result in large data sets. These data purport to be entirely objective, well within line with Frederick W. Taylor’s principles of *Scientific Management*, espoused in his book of the same name [224]. The technology of tracking devices has grown and has changed fieldwork for many researchers. Instead of the long hours following a species and taking notes of its behavior and micro-movements and activities, the animal is captured and fitted with the apparatus and then tracking data can be downloaded. This is a very different kind of fieldwork than Konrad Lorenz or Jane Goodall had practiced: patiently observe, allow animals (incl. birds) to habituate to the researcher’s presence so that natural behavior can be scored and reported. Their studies were invaluable because they revealed important aspects of natural behavior, social dynamics, daily activities, and other behavior.

The new technology, in a way, does the opposite. It not only alienates the researcher from fieldwork as a sharing of the life of the animals one observes, it also invites invasive procedures and interventions that may actually stop natural behavior. Now fieldwork may often consist of just catching wild birds, fitting the apparatus, then letting the animals go, and, after short interactions with the reluctant target birds, return to the computer and download the data provided by the tracking device.

Doing this has given fieldwork a boost. This is true of marine life and mammals: Large numbers of movements could be recorded and, for conservation of vulnerable migratory birds, having data for their migration path enables protection of these paths and stop-over sites [225,226,227]. Nobody doubts the importance of such in situ research. There are alternatives, however, to fitting the birds with devices, such as radar aeroecology [228] or simply taking photographs at various points of the route for identification [229]. For research not involving migration, telemetry cannot lead to a deeper understanding of the natural behavior of species studied, the kind that is important for successful zoo management and for reintroductions.

While telemetry is now very well established and its technological advances are still evolving, specific concerns about its application to birds, as will be raised and cited here in numerous papers, have barely been heeded. From the studies presented here we need to ask whether this technology is more harmful for some species than for others, which models are less damaging, and which time frames for having such attachments should be a maximum limit and why. The data so far suggest that, for birds at least, this technology may be counterproductive or dangerous. It is the task of animal welfare to minimize harm and, as far as can be ascertained, this does not appear to be a major concern or at least not considered a high priority in an increasing number of avian field studies.

The answer to the question as to why researchers may have turned a blind eye to the negative effect of devices on birds may lie partly in the adulation of technology possibly overriding concerns for their welfare. N. Postman wrote a book in 2011, called *Technopoly: the surrender of culture to technology* [230]. He argues that ‘technopoly’ is founded on the belief that technology is superior to complex and variable human thinking and judgement. This, in his view, is in keeping with one of Frederick Winslow Taylor’s *Principles of Scientific Management*. These are efficiency, precision, and objectivity. Importantly, Postman also argued that technology does not invite a close examination of its own consequences [230]. In a wider sense, he argues that the new gadgets *create a culture without moral foundation* [230].

Postman’s words can be directly applied to much research involving the use of telemetry in bird studies. Those who have been swayed by the ease of this new technology will have a strong counterargument, well represented by Kavelaars and colleagues [231], who summed up its benefits for bird studies in the following manner, in direct contrast to Postman:

“Recent technological advances facilitated the continuous improvement of avian-tracking devices allowing the study of individual movement patterns in ever-increasing detail. The emergence of cutting-edge tracking devices caused great leaps in the study of movement ecology in the past couple of decades, thereby increasing our knowledge about the global space-use of wide-ranging birds”.

[231]

The language used in describing telemetry could not be captured better than in this article: The ‘recent technological advances’, ‘continuous improvement’, and ‘cutting-edge tracking devices’ are key words that engender a sense of ‘trust’ and even excitement. They imply the promise of high returns, of producing very useful and objective data (i.e., ‘what really happens’) previously not within reach of human knowledge or observation. Wisely, Kavelaars et al. limited their observations to migration/air space use in which such devices may be more defensible (perhaps not in the current designs) than in other contexts.

Technology, as a savior and unassailable friend, as Postman had argued [230], allows for a good deal of ‘collateral damage’ that may be tolerated, considered a small price to pay for large data sets, or it goes unnoticed. Ethical concerns about collateral damage, such as profound damage to health, reduced nesting success, and even risks to survival of birds studied in their natural environment ought to be at the forefront of experimental design. Questions about the equipment are largely concerned with the length of time such equipment functions, how long the batteries last, and how well the item remains intact on the animal. While pragmatic, what should be asked is what effect the equipment might have (1) on the organism’s well-being and overall health, (2) on its sense of direction, (3) on its skills for finding food, and (4) on reproductive behavior. The literature so far suggests that there can be effects in one or more of these categories. Producing substantial data sets via technology may look fine but may also introduce substantial distortions and errors. Pain, fear, or discomfort change the behavior of organisms [74,232,233,234]; so, the question arises: how well one can trust the data produced?

In publishing results, one may well notice the omission of anything that could sound remotely emotive or raise suspicions over the welfare implications of the research. Some of the subjects that are part of such technology-driven short- or long-term field studies may well suffer, be subject to chronic pain, or even die. For instance, lowered nest success may not mean that eggs were infertile but that nestlings starved to death because the parents (at least one of whom was tagged) could not provide enough food. Lowered ‘return rates’ imply that not all animals were recovered after the experiment, and one could translate this as actually meaning ‘likelihood to have perished’. Possibly, the enthusiasm for this technology overrode other/empathetic concerns, as Postman had suggested [230]. These potential outcomes, as will be described below, make such devices a very real but so far somewhat rather uncontested and often off-the-radar welfare issue.

### 3.3. Some Harmful Effects of Telemetry

A decade ago, it was already clear that negative transmitter effects were known and widespread. Hill and Elphick then reported negative transmitter impacts [234] for more than 63 passerine species. These included entanglement with vegetation or body parts. Non-entanglement-related injuries affected 27% and 19% of species, respectively. Significantly, they found that out of 60 researchers only two researchers had documented these problems in the peer-reviewed literature [234]. This is just one example of many indicating that, although much of the problem is hidden, it is real and serious.

**Figure 3:**
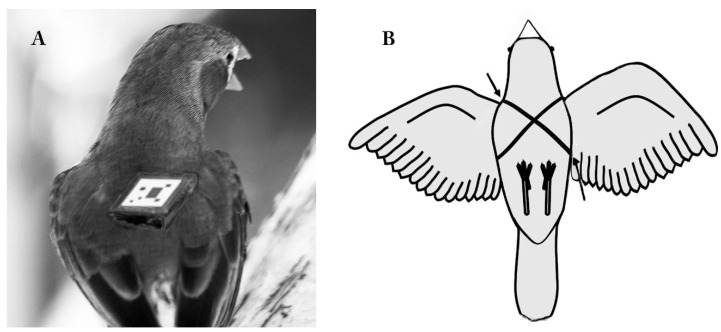
An automated barcode tracking system designed for behavioral studies of birds. (**A**) How an attached unit is affixed on a zebra finch’s back (adapted from Alarcón-Nieto et al., 2018 [238]). (**B**) Ventral view of the harness that holds the barcode device Note, the arrows facing down and up indicate particularly risky areas for inflammation, infection, or damage to skin or wings. (Photocredit: G.Kaplan- bar code is superimposed; approx.to scale).

To examine how these harmful effects occur, the harnessing has been shown as a possible cause for discomfort, inflammation, or infections [235]. Note in Figure 3B the harness overlaps across the chest and is affixed to the front and the back in such a way that the string is right next to the front and the rear of the wing. In front, it can cut into the patagium, one of the most important adaptations in birds to enable flight (and gliding in some lizards and bats). It links shoulder and wrist bones and takes the pressure of air forming the ‘sail’ that enables flight. The patagium is also the ‘Achilles heel’ of bird flight: once inflamed or cut, it cannot regrow or be repaired and disables the bird permanently, making any future flight impossible. There are different styles of flying, the gliding, the low and high wing loading, the slow wing movements versus the fast. Diving seabirds have a particularly high wing-loading and use energetically expensive continuous flapping flight [236,237]. There may be slight differences in how harnesses or wing tags may affect birds, depending on flight style but this has not been researched. However, it is clear that harnesses with backpacks may do damage (Figure 3B).

**Figure 4:**
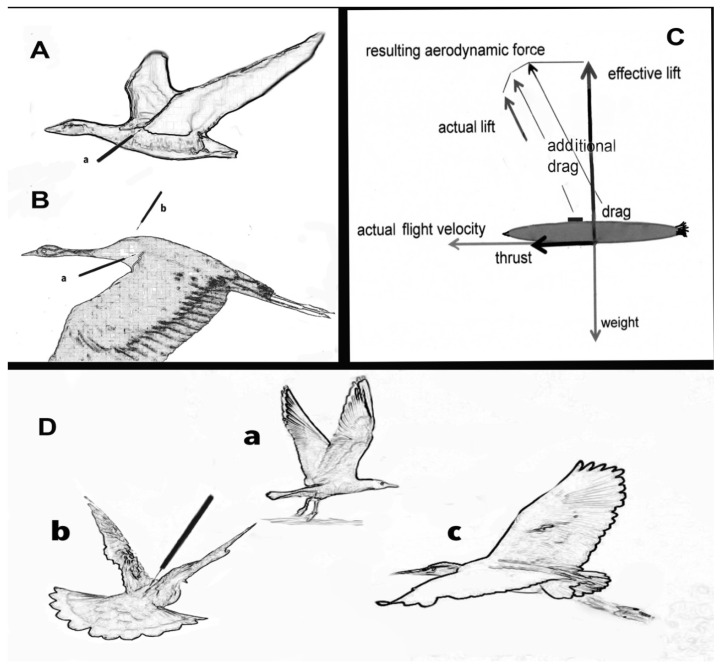
Flight positions. (**A**) Upward movement (**a**): arrow points to pressure point of harness cutting into edge of wing. (**B**) Smooth downward movement (**b**): where device would be attached. (**C**) Diagrammatic presentation of the body of a bird (excl. wings): the process of lift and thrust and the aerodynamic forces that determine actual lift and velocity, showing how the device (black box, dorsal position) creates extra drag. (**D**) (**a**–**c**) showing different flight positions; in (**b**) arrow indicates how the device interferes with the upward swing; in some species it can even prevent the full upward movement of the wings and thus make the lift more difficult; in (**c**) there would be least wing contact with a back-fitted device but maximum drag.

Equally, the position of the device, typically placed on the back of the bird, tends to be problematic. Flight consists of wing movements that create both lift (down swing) and thrust (upswing). The air moves over the body and over the back in such a way that it causes minimal drag and turbulence. Figure 4A,B (up–down swing) below shows the perfect aerodynamic shape of an avian body in flight. The legs are tucked in backwards and everything in its smooth shape works to limit drag and turbulence. With a device attached (Figure 4C) it is not just that drag is created and energy costs increase but the harness is pulled back into the wing by the force of the air from the front. Studies by Croll et al. [239,240] showed, already 30 years ago, that devices such as those described here affect drag and wing-loading and increase the energy required for flying (and diving) [239]. These attachments will render the bird far less efficient in flight and may disadvantage the bird if a predator pursues it, not to mention the possible strain on the bird, its stress levels, and even distress when unable to control its flight as well as it is used to be able to do. If flight or diving is part of foraging, the harness and attachment are particularly detrimental and may result in reduced efficiency in foraging, i.e., lead to weight loss.

There are other important social impediments, which I believe are also of importance in delivering appropriate standards of welfare. The appearance of the back of a bird is partly an indication of its health. The uropygial gland, placed at the base of the tail feathers, has a great deal to do with the overall health and good appearance of plumage [241]. The uropygial gland is important in the defense against the growth of fungi, chewing lice, and ectoparasites, which mainly feed on feather keratin. Depending on the degree of infestation, they can cause rapid feather deterioration and affect a bird’s overall fitness but, it seems, the cocktail of fatty acids and long-chain alcohols of the preen gland limit the effects and success of ectoparasites [242]. The affixed device makes reaching the preen gland a good deal more difficult for the bird, and the device itself can become a refuge for ectoparasites and especially the growth of fungi (Figure 3A).

Little imagination is needed to see or at least suspect that the device affects daily activities as well as flight (additional energy expenditure because of an attached device that indisputably interferes with aerodynamics). There is a further reason for concern. A barcode marker on the back of a bird is highly conspicuous and certainly not helpful in terms of camouflage and survival. For females, such barcodes on the back are particularly damaging for reproduction. Males mount females when copulating, but may not do so with such a dramatic set of dots and unfamiliar structure on her back, as shown in Figure 3A. These foreign bodies could mitigate against any mating and bonding attempts altogether. One would need to score the behavior of a group or pair of zebra finches to see whether or how the social dynamics change once such a glaringly obvious attachment. Is fitted.

Finally, some of such attachments have been fitted subcutaneously. We are used to cats and dogs being fitted with microchips in a small area at the back of the neck. With some exceptions [243], there tend to be no problems with such a subcutaneous attachment in companion animals. The chip is very small relative to the size of the neck and body and, importantly, mammals have an epidermis that is not only pliable but strong. The skin can be lifted quite readily.

In birds, the actual skin, except in some diving birds such as penguins, is paper thin and brittle. Just administering a simple saline solution subcutaneously is a difficult task because even the smallest insertion can tear or puncture the skin and let the fluid dribble out again. Hence, a subcutaneous ‘anchoring’ can only mean that the device is anchored in the muscle. One study of Xantus’s murrelets, *Synthliboramphus hypoleucus*, admitted that this is the case [244]. The authors suggested sedation using isoflurane inhalant anesthetic to reduce the pain and stress associated with attaching the GPS device. They also suggested that, by doing so, the trauma of pain and handling would be reduced. They cited Heatley et al. [245] in whose paper it was reported that corticosterone (stress hormone) concentrations in manually restrained Amazon parrots, *Amazona ventralis*, were significantly higher than for birds anesthetized using isoflurane. However, then the authors dismissed the traumatic event as a ‘once in a lifetime experience’ that would ultimately ‘not be worse than other stressful events in a bird’s’ life [245]. This is unlikely to be the case since the anesthetic wears off and the device stays on. It most certainly will interfere with the bird’s daily foraging, preening, and flying activities. For the bird, it is, thus, not a “once in a lifetime event” but just the beginning of a potentially traumatic and persistently painful chapter in its life [246].

Barron and colleagues reported as long ago as 2010 that there are deleterious consequences for birds having to carry devices and transmitters which, in their minds, also affected the validity of data collected, i.e., resulting in bias [220]. They distinguished between methods of attachment, such as backpack/harness, collars, glued and taped, subcutaneous anchoring, implantations, or attachment to breast, wing, or tail. They found that birds with devices secured by a subcutaneous anchor-shaped wire had the lowest nest success, and next were birds with a harness attachment [221]. In another study of reproductive success in 25 adult tufted puffins, *Fratercula cirrhata*, in Alaska [247], the authors confirmed that those with subcutaneous radio markings produced low-weight offspring and stunningly lower fledging rates (33% vs. 84%) than did other birds without transmitters [247].

It is argued here that these kinds of procedures for the sake of a few extra data are unethical and incompatible with new welfare thinking. It is also suggested here that stressed birds and/or those in pain do not behave naturally and the data produced from such contexts are not data that one can trust, and thus not good science as has been argued time and again [245,246,247].

If some of the studies are designed to save declining populations, it would seem more than just counterintuitive to use methods that hasten the species’ decline. On those grounds alone, the method of attachments to get data should be regarded as questionable if not unacceptable. On welfare grounds, even though the evidence is indirect (i.e., the effect on the parents was not directly examined but the tagged birds are likely to have poorer foraging success), the methodology should not be acceptable. A paper by Thaxter and colleagues [248] reported on the responses of black-backed gull, *Larus fuscus*, and great skua, *Stercorarius skua*, to a GPS device attached using a crossover wing harness and found that the two species responded differently to the same devices. There was little measurable difference in behavior in the gulls pre- and post-fitting but, in great skuas, there was strong evidence of reduced overwinter return rates, which, in most cases, meant that they had died.

A convincing study presented the effect of the devices on a flightless rail species, takahe, *Porphyrio mantelli*, of New Zealand [249]. Much of my argument above rested on the assumption that the main criterion for the negative impact of telemetry had to do with flight. By examining the effect of the devices on a flightless bird, the researchers demonstrated that the effects are far more fundamental. They showed that daily energy expenditure increased by 8.5% when the takahe were carrying radio tags. They concluded that a greater thermoregulatory effort was likely to be the main factor involved, which “could potentially reduce survival in this endangered species, particularly in its montane habitat in winter” [249].

It is likely that this extra thermoregulatory effort was not just related to the size and position but also to the weight of the device. To date, for instance, the Animal Ethics Infolink of 2020 suggests that >5% of a bird’s body weight is the preferred option for strap-on devices [250]. While this weight sounds reasonably low, a quick check, translating this figure of 5% of body weight into an average weight of a human male (say 80 kg), would mean the weight of 4 kg affixed day and night. This is substantial and clearly a burden to bear. It is easy to imagine that even in humans, over time, this would have implications for posture, well-being, sleep pattern, and indeed, overall health.

Wing or patagial tags, whether put on flighted or non-flighted birds, have also had negative reports and one may add to the list a range of diving birds. Studying penguin diving behavior, researchers found that biologgers or flipper tags negatively affected diving behavior and overall health [251,252]. In a study on magnificent frigatebirds (*Fregata magnificens*) in Barbuda in the West Indies (Eastern Caribbean), wing-tags were studied as an alternative to backpacks and harnesses. The researchers rightly raised concerns about the nearly three-fold difference in percent nest success of wing-tagged versus control nests [236].

Invasiveness in study methods is not limited to attachments. As mentioned in the introduction, field studies regularly include a variety of capture methods such as traps (mist nets, cannon and rockets nets), funnel traps, or specific traps at nest sites. Additionally, in order to continue observing birds in the wild, captured individuals tend to be marked in some way, be this with one or several of the various leg bands (color or metal), leg tags, radio or satellite transmitter, wing markings, neck collars, nasal discs and saddles (the latter two for waterfowl), or dyes or ultraviolet markers [11]. Even some of the seemingly more benign leg bands can inflict injuries including color banding (plastic) on legs [253].

Given the volume of studies reporting negative outcomes, one can only conclude that attachments of any kind ought to be considered very carefully and, I suspect, in most cases evidence might well indicate that they are in breach of the basic rule of welfare: Do no harm.

Invasiveness and interference in birds’ lives do not depend on, or end with, attachments. Research may require return nest site visits, capture or local examination of nestlings for weight, blood sampling, and the like. These alone can have detrimental effects on birds, as was confirmed in studies of yellow-billed and Pacific loons (family Gaviidae) [254].

Based on such damning and detailed existing evidence, one cannot but express surprise why many of the devices used in field studies continue to be sanctioned and supported by institutions and facilities granting project and research permits and by journals accepting papers based on research relying on such devices. Ironically, in many of these projects, as mentioned before, the stated intention is conservation.

Zemanova’s point, raised before [176], is worth reiterating here, namely, that we need to move from invasive to non-invasive methods, employing our imagination to move to more compassionate methods of wildlife research [176]. Vulnerable or endangered species are more likely to be targeted for field studies involving the use of harnessed devices and other attachments. As S. Michael and colleagues convincingly showed in New Zealand’s endangered takahe [235], attachment of devices could potentially further reduce survival in this endangered species. It makes little sense to me to engage in alleged conservation activities for a species that might actually accelerate its decline.

Alternative methodologies have been explored to replace tracking devices but nowhere near the extent to which alternatives for blood sampling have been proposed and used effectively as already described. Finding alternatives for GPS tagging has tended to be more concentrated on mammals [255]. One avian study on southern cassowaries innovatively used visual lures in order to attract these shy but very curious and very large flightless birds into the range of camera traps [256].

From a welfare point of view, it is of less consequence whether a species is endangered or not: The mere practice of research that inflicts harm and suffering on birds, be this in the field or in the laboratory, just for the sake of getting data in a more convenient and time-saving manner, probably would and should be regarded as unacceptable.

## 4. Solutions

Based on existing evidence, it would seem to be an urgently needed task to review any assumptions, actions, or practices, be they in zoos, in zoo-initiated captive breeding programs, or in field studies from the point of view of avian welfare. Substantial and binding changes ought to be made to achieve standards of welfare that begin to look comparable to modern welfare standards already achieved. Most importantly, such new standards ought to be made relevant and effective for birds. I shall give a few examples here to make the point that some of these changes are easier in practice than it appears, at least for legal/administrative/regulatory levels of implementation.

There are many solutions possible merely by taking some proactive legal and welfare steps at the highest level of local, professional, scientific, and government associations and journals, from the point of view of Animal Welfare. Additionally, I am suggesting that the widely acceptable research practices of the use of attachable devices are more closely examined by Animal Welfare committees.

In in situ conservation, camera traps are a very valuable tool for field-studies aimed at establishing animal activities in a specified area and this is by and large non-invasive [257,258]. A very useful paper by Randler and Kalb compared different camera traps specifically designed for birds at different distances and indicated also the best settings [259]. Importantly, the human observers were there, at the same time, scoring the presence of birds and their numbers and then comparing the results with those acquired from the camera traps. By using this method, they could determine which cameras worked best/gave most reliable data and at what distance. Hence, there are techniques that need to be acquired first by the researchers to make camera traps work in a way that can reliably support research. In one set of experiments, the researchers used a food incentive to get birds into camera range, in another, the environment remained entirely unmanipulated and they still acquired publishable data [259,260]. This is the kind of technology that is non-invasive and should be fostered. Collecting images is perhaps not as attractive as other electronic devices are in terms of ease of gaining data. Trackers produce ready-made data that can then be put directly into a statistics program, hence provide a set of results. In camera traps, the scoring is still up to the researcher, translating the visual information into data. Of course, this takes longer but that is not a sound reason for choosing one method over another. Research takes as long as it takes, and time budgets can be integrated into research projects: Shortcuts that compromise welfare standards should not even be a consideration.

### 4.1. Telemetry and Ex Situ Conservation

(1) The manufacture of telemetry items for research: I am aware that the manufacturers of these devices are not to blame although their products may vary from relatively innocuous to dangerous. The problem lies fairly and squarely with the institutions giving permission to individual researchers to use specific tools for their research and some blame must also go to the researchers themselves who could express concerns and could report these to manufacturers directly, as surprisingly few may have done. Equally, journals can play their part by not just accepting an ethics approval statement from the institution of the researcher but by making their own separate ethics statements that preclude field practices known to be harmful.
(2) We need more research of the effects of these devices on the animals’ behavior. For example, some avian species, resident or migratory, may be affected in different ways. I am not aware that there has been detailed research conducted on the effects of signal transmission although we know of birds detecting electromagnetic fields [260].
(3) The AAV (American Avian Veterinarians), as was shown above, with its position statement has had a powerful effect on some zoo practices. It could easily add another position statement, such as: that it does not support any procedures that attach a foreign object to a bird on a temporary or prolonged basis unless it can be shown that such attachment has few if any deleterious effects that would impact on overall health, cause prolonged pain or permanently and irrevocably alter avian normal function or even result in death. (author’s wording).

That alone could help reset thinking about attachments to birds, save lives and reduce any unnecessary suffering, reduction of offspring or lifespan.

There is a place in international law including more compassionate and explicit statements about the way animals can be studied in the wild [261].

### 4.2. Ex Situ Conservation and the Problem of the Technology Employed

The thousands of studies now using tracking devices on birds have become a major welfare issue [262]. To address the problem, some papers have proposed non-invasive alternatives, some of which have been described here. While, admittedly, some of these alternatives work better on mammals and less well on migratory birds, there is room for regulations to be implemented, be these for bird organizations, research institutes, and universities and its relevant overseeing bodies that approve and fund research projects nationally. These could include:

(1) limiting the amount of time such devices (no matter how improved in weight) should stay on a bird;
(2) ensuring to never use devices on females in any studies, and most decisively not around breeding time;
(3) abolishing practices/devices that lower reproductive rates, have been shown to lead to serious physical health issues, long term suffering or death in a known percentage of birds or in specific species.
(4) explore the possibility of smaller and more aerodynamically shaped devices, more in the shape of pencils rather than in the shape of small match boxes.
(5) ensuring that no device is on any bird beyond a set period of time (the shorter the better) and that there are safe and predictable ways in which such devices will self-release/self-destruct; at the moment it is not always clear how and when such devices, once fitted, will actually be removed (and by what means).
(6) with the decline of migratory birds, in particular, there is a heightened need to learn where their flight paths are so that such flight paths can be protected. Under such circumstance it would be twice as important to go back to manufacturers and seek innovation of new and significantly smaller and lighter tracking devices that are tested extensively and meet new standards, i.e., are proven not to cause friction in flight or damage wings or other parts of the body or leave them so weak that they cannot complete their, often arduous, migration flights [222].

Clearly, in all projects on avian species, alternative techniques should be explored and ways found to be non-invasive (as camera traps mentioned above are, they are already used widely and effectively to locate mammals and birds). There may not always be useful alternatives, but the convenience of this modern technology has perhaps persuaded individual researchers to not even ask questions related to any of the three Rs [263,264].

The 3Rs should also be further investigated leading away from invasive techniques. The use of feces in capercaillies, *Tetrao urogallus*, was already mentioned [182,183]. Non-invasive genetic sampling for estimates of numbers and population structures in the same species have now been successfully explored in capercaillie [265] as well as in large macaw species (such as *Ara macao* and *A.chloropterus*) [184]. Non-invasive photo-identification techniques have been employed widely in aquatic animals and for a considerable time, also involving citizen science participation [266] involving people who send images of sightings and GPS detail to be collected on large data bases. This has allowed identification of sharks, whales, and much smaller aquatic animals. Very similar techniques have been used in amphibians, reptiles, and mammals [256] but, for birds, non-invasive photo-identification on a wider scale is a relatively recent consideration and now involves adults [267,268] as well as nestlings [269]. Hence, a range of alternatives to invasive methods is already available, reiterating a point Bekoff made nearly 20 years ago: Ethics *is* important in conservation biology [270].

### 4.3. The Zoo Environment and Ex Situ Conservation

It is heartening to see that some leading zoos have switched to evidence-based enclosure design and provision of species-specific behavioral husbandry and have stopped many of the reprehensible practices and surgical interventions. This alone is not enough. Ex situ conservation usually has the explicit aim of reintroductions into the wild and the problems in these reintroductions cannot be overlooked. Likewise, field studies often supporting or undertaken in conjunction with captive breeding programs require close assessment and in some of the improvements, new welfare standards can play an important role. One way to achieve this, as was recently suggested, is to merge animal welfare regulations between agricultural, laboratory, and zoo environments [271] and, one should add, extend this welfare net to ex situ practices.

Indeed, the current compartmentalization of animal welfare according to industry with varying standards of enforcement has led to big holes in the welfare model. The net has to be cast wider and be made tighter than it is now. It needs to include clear regulations for researchers and any institutions underwriting field research to gradually eliminate as far as possible any procedures, invasive actions, handlings, and devices that lead to injury, infection, weight loss, reproductive failures, shorter life spans, or disappearance (presumed dead). Alternatives should always be considered not only in terms of the 3 Rs but in terms of finding ways of collecting information without causing stress, distress, or pain to the species under investigation. In some respects, this proposal here requires a new rigor in the application of animal welfare standards to overcome the status quo that has clearly permitted many breaches in animal welfare standards and, unacceptably, without much rebuke or repercussions [272].

## 5. Conclusions

To achieve a convergence of animal welfare regulations and better welfare outcomes across a wide field of endeavors may seem ambitious but some such attention may require no more than including extra statements in already existing sets of Animal Welfare regulations.

There are many, seemingly small, ways to improve avian survival. Animal welfare can play a role in steering researchers away from harmful technologies to non-invasive ways, showing innovations in preventing harm to birds, as was argued throughout the paper. An organization called ‘Defenders of Wildlife’ has suggested stronger protection for migratory birds and involving the legislature in conjunction with welfare agencies to come up with new legislation and wildlife corridors that would keep wildlife safe. Their mission is to protect and restore imperiled species “by transforming policies and institutions and by promoting innovative solutions” [272]. Indeed, these are called for in the domains covered in this paper. Some technology used in research can be replaced by employing the 3 ’s [264].

The problems concerning invasive techniques and technologies have been noted for well over two decades, without notable change. There are some light-weight products of data-loggers on the market, which are apparently less damaging to the wearers, but this was tested only in shearwaters [273]. Another study showed that corticosterone levels rose when common murres *Uria aalge* and thick-billed murres *U*. *lomvia* were fitted with ‘small geolocators’, but that they survived [274]. Despite the honesty in showing direct effects of the geolocators, one cannot take too much comfort in the reassurance that mere survival is the (extreme) yard stick for acceptable standards in animal welfare.

If anything has changed, the use of harmful tracking devices has increased in numbers and frequency across projects. Indeed, tracking devices have become more or less common practice as if their use were the only way to do science, and a guarantee that ‘real’ science was being conducted. Geen and colleagues [263], in their survey of telemetry, noted that less than 50% make any detailed reference to the technology used and even fewer have included possible negative health effects. They also noted with some concern an actually and steady increase of 4.4%. in publications using tracking devices, calling for more systematic documentation of potential effects in peer-reviewed publications in order:

(1) to support more rigorous science and
(2) to further improve bird welfare [263].

The current state of affairs would not be considered acceptable if we applied to some zoos and ex-situ conservation research activities the same welfare standards as used in laboratories, domestic animal care, and in the farming industry. One may also conceive of calling on manufacturers to alter present designs (and test them) to reduce or eliminate painful experiences that can mar natural behavior of birds, or even be fatal. Journals can play a role in raising standards in animal welfare in wildlife studies generally by insisting on a full description not only of methods used but of justification of methods and a detailed description of invasive procedures, how invasive procedures were minimized or replaced.

Some researchers might argue that the question of doing harm in birds is a marginal issue because it is all done for the greater good of species conservation (the risks/benefit argument). Perhaps a moment of reflection is needed here: The bird numbers involved in being harmed in some way is substantial, be this in mismanaged birds in captivity, in failed reintroductions and field research methods. After all, Geen and colleagues [262] had surveyed more than 3400 recent publications using telemetry, as said above. Even if each project only tagged 10 birds, this would amount to close to 34,000 birds. If we talk about numbers in zoos (especially smaller private facilities), the numbers rise to hundreds of thousands, possibly millions.

The problem is thus systemic rather than isolated and needs to be fixed via education, focused training, and via new welfare rule interventions. Welfare standards have improved substantially over the years in some countries but the shift to urgent conservation needs and the advances in technology have also set new challenges for animal welfare generally, and for Class Aves specifically.

In terms of ‘good science’, one cannot stress enough that fear, pain, hunger, distress, weight loss, and any number of assaults on the health of an organism change behavior and distort results, at times significantly.

The challenge here is to convince researchers and staff dealing with birds generally that (1) birds have the entire range of emotions and physiological responses as other mammals (as argued in the first section of this paper) and (2) that negative experiences, be they physical or emotional or both, may have serious and long-term negative effects and outcomes. Finally, (3) that it may take training to identify signs of ill-health (physical or mental) because of the avian ability to mask such signs of weakness. Training people not just in ecology but in avian ethology would help in dealing effectively with birds in zoos and some ex-situ conservation programs, including giving birds the opportunity to acquire essential skills before release attempts are made. Young birds have to learn a great deal, as outlined in this paper, before they can succeed in the wild. In the last decades, some 10% of zoos have been able to increase welfare standards by linking up with international bodies and appropriate specialists [123,124], clearly showing significant progress but an alarming number of field studies seem to have almost gone in the opposite direction. Hence, as regards the trends in technology in field research, animal welfare agencies and research institutions might need to step up, too, and sooner rather than later [122].

## Figures and Tables

**Table 1:** Common Behavioral Problems in Caged Birds (of several orders).

Needs (Physical)	Natural Habits	Captivity	Potential Outcomes Challenges Solutions			Sources
**1. Foraging**	Ground/arboreal	Most food in single bowl	Boredom, lack of motivation, feather picking	work for food	Food search/Food variety	Lindenmayer et al. 1996 [28]; Grindlinger and Ramsey 1991 [29]; Meehan et al. 2003 [30]; Marino 2018 [31]
**2. Flight**	In most species, flight is a daily activity	Usually impossible	Muscle atrophy	Provide opportunity and motivation	Allow for regular exercise	Gaunt et al. 1990 [32]
**3. Light**	natural	Great variation	stress	Avoiding artificial light	Avoid harsh and direct light by providing half dark corners	Mellor et al.2018 [33]
**4. Sleep**	Birds are the only animals, outside of mammals, known to engage in slow wave sleep and REM sleep	Problem if kept in lit living rooms after dark or kept awake by sudden noises and bright or flashing lights	Aggression; Sleep deprivation; Loss of appetite	Non-threateningEnvironment without artificial light	Finding a warm and quiet spot with suitable night light	Lesku et al. 2011 [34]; Cooper et al. 2019; [35]; Cornelius et al. 2018 [36]; Hodinka et al. 2020 [37]
**5. Predictability**	Daily routines	unpredictability	loss of control	Taking time tabling seriously	Good time tabling of extra activities	McMillan 2005 [38]
**6. Nutritional needs**	All four food groups	Commonly deficient	Depression, weight loss, fractures, even vomiting and diarrhea	Creating variety	Early training in what is edible	Fisher 2013 [39]
**7. Noise**	Forest and plains levels of sounds	Noisy people, radio, television, and especially ultrasonic or computer sounds	Stress, fear, shock	Avoiding sudden noises and nearness to noise-producingtechnology	Reducing the noise and removing the source	Baldwin et al. 2007 [40]
**8. Parental care**	Substantial; Time involvement	Deprivation of parent care	Effect on neural development and adult functioning. Increased anxiety, impulsivity, aggression, and behavioral abnormalities such as motor stereotypies	Justification for separation now doubted	Absence of parental care has no panacea, but negative effects can be mitigated by providing mentors of the same species and/or environmental enrichment	Aengus and Millam 1999 [41]; Feenders and Bateson 2013 [42]; Mason and Rushen 2008 [43]; Greenwell and Montrose 2017 [44]
**9. Attachments**	Within pairs or flocks, pairs remain in close spatial contact	Single, or mismatched; Multi-species	Depression, Physical signs of illness	Companion/buddy System	Companion/buddy System; Isosexual pair housing	South and Pruett-Jones 2000 [45]; Doane and Qualkinbush 1994 [46]; Meehan et al. 2003 [43]; Duque et al. 2020 [47].
**10. Variety**	natural	Lack thereof	Less evidence	To make variety species appropriate	Increasing meaningful variety	Seibert 2020 [22]
**11. Personality**	Vastly Different	Ignored too often	A range of abnormal behavior, incl. screaming, aggression	Important to first establish compatibility in multi-housing	Let birds choose their partners by themselves	Zeigler-Hill and Highfill 2017 [48]; Richter and Hintze 2019 [49]
**12. Cognitive needs/brain function**	daily life stimulation, problem solving and decision making	of increasing importance	Not much known other than effects on ‘mood’	A good deal known about corvids and parrots but assessing cognitive needs is far more difficult	Can only be assessed via behavior and that presupposes knowing the cognitive skills very precisely	Bateson and Matheson 2007 [24]; Clark 2017 [50]; Hopper 2017 [51]; Rogers and Kaplan 2019 [52]
**13. Knowledge**	Learning from mentors, parents, experience	Limited to captive environment	Unsuitable for release if in a captive breedingprogram	Provide challenges (animal agency)	Exposure to trial-and-error tasks	Spinka and Wemelsfelder 2018 [53]
**14. Experience stimulation**	varied	limited	Boredom	Expand environment	Stimulation is sensory or environmental but can also be social	Pepperberg 1987 [54]; 1994 [55]; Evans 2001 [56]; Swendsen 2019 [57]
**15. Problem-solving**	regular	---	Birds in research facilities often get set tasks of this kind and are often better off than captive birds without such stimulation	To re-engage the individual with its environment	Often successfully used by making food more difficult to access, exploiting skills the species is known to have	Auersperg et al. 2018 [58]; Rössler et al. 2020 [59]; Laschober et al. 2021 [60]
**16. Exploration**	daily	limited	Stop locomotion	Prevent depression	Create and change areas that lend themselves to exploration, such as tree segments with loose bark	Mettke-Hofmann et al. 2002 [61]
**17. Danger, approach to novelty**	Recognizing danger/neophobia	loss of curiosity or loss of interest	Fear, anxiety, or indifference (non-recognition)	Settings conducive to avoid visual constants that might indicate danger	Places for hiding and height to flee to	Mettke-Hofmann et al. 2002 [61]; Papini et al. 2019 [62]

## Data Availability

The data are contained in the cited references.
